# The miR-216/miR-217 Cluster Regulates Lipid Metabolism in Laying Hens With Fatty Liver Syndrome *via* PPAR/SREBP Signaling Pathway

**DOI:** 10.3389/fvets.2022.913841

**Published:** 2022-05-31

**Authors:** Lihui Zhu, Rongrong Liao, Jiwen Huang, Huaxiang Yan, Changfeng Xiao, Yunzhou Yang, Huiying Wang, Changsuo Yang

**Affiliations:** ^1^Institute of Animal Husbandry and Veterinary Science, Shanghai Academy of Agricultural Sciences, Shanghai, China; ^2^National Poultry Research Center for Engineering and Technology, Shanghai, China; ^3^College of Animal Science and Technology, Zhejiang Agriculture and Forestry University, Hangzhou, China

**Keywords:** laying hens, fatty liver, miR-216/miR-217 cluster, lipid metabolism, fat deposition, interventional strategy

## Abstract

Fatty liver syndrome (FLS), a common metabolic disease in laying hens, caused by excessive hepatic fat deposition is a bottleneck in the poultry industry. However, no specific therapeutic methods have been developed. Evidence suggests that microRNAs (miRNAs) are essential for liver lipid metabolism and homeostasis, providing strong evidence for targeting miRNAs as a potential treatment option for liver diseases. However, the roles of miRNAs in the pathogenesis of FLS remain unclear. In present study, RNA-sequencing was performed to discern the expression patterns of miRNAs in normal and fatty livers of laying hens. In total, 12 dysregulated miRNAs (2 down-regulated and 10 up-regulated) were detected between the normal and fatty livers. Functional enrichment analysis showed the potential impacts of the dysregulated miRNAs on lipid metabolism. Notably, miR-216a/b and miR-217-5p, which belong to the miR-216/miR-217 cluster, were up-regulated in the sera and livers of FLS chickens, as well as free fatty acid (FFA)-induced LMH cells. Oil-red O staining revealed that up-regulation of the miR-216/miR-217 cluster induced lipid accumulation in FFA-induced LMH cells. Furthermore, the dual luciferase gene reporter assay and RT-qPCR analysis demonstrated that 3-hydroxyacyl-CoA dehydratase 2, F-box protein 8, and transmembrane 9 superfamily member 3 (*TM9SF3*) were directly targeted by miR-216a/b and miR-217-5p, respectively, and suppressed in the fatty livers of laying hens. Moreover, overexpression of the miR-216/miR-217 cluster or reduction in *TM9SF3* levels led to activation of the proliferator-activated receptor/sterol regulatory-element binding protein (PPAR/SREBP) pathway. Overall, these results demonstrate that the miR-216/miR-217 cluster regulates lipid metabolism in laying hens with FLS, which should prove helpful in the development of new interventional strategies.

## Introduction

Fatty liver syndrome (FLS), a common metabolic disease and the most frequent non-infectious cause of mortality in laying hens, is characterized by excess deposition of triglycerides (TGs) in hepatocytes due to an imbalance between hepatic lipogenesis and fatty acid (FA) oxidation, resulting in reduced egg production and death (1–3). However, no specific therapeutic methods have been developed after decades of research. Increasing evidence indicates that miRNAs associated with lipid metabolism are frequently dysregulated in human non-alcoholic fatty liver disease (NAFLD) (4). For example, inhibition of miR-21 through RNA interference was reported to suppress synthesis of TGs (5). Moreover, inhibition of miR-122 suppresses lipogenesis via targeting Sirtuin 1 (6), while down-regulation of miR-34a increases the expression of proliferator-activated receptor α (*PPAR*α) and several target genes of *PPAR*α, suggesting that miR-34a is involved in regulation of lipid metabolism (7), and hepatocyte-specific inactivation of miR-379 reduced the concentration of plasma TGs in healthy mice (8). These results provide strong evidence for targeting miRNAs as a potential treatment option for NAFLD.

Similar to NAFLD, dysregulation of miRNAs associated with lipid metabolism has been reported in the livers of commercial caged laying hens. For example, miR-122, which targets the lipid metabolism-related gene fatty acid-binding protein 5, and miR-101-2-5p, which targets the lipid transporter apolipoprotein B, are reportedly highly expressed in the chicken liver (9, 10), while miR-33 negatively regulates the lipid oxidation regulator gene carnitine O-octanoyltransferase (11). Moreover, overexpression of miR-34a-5p, which targets acyl-CoA synthetase long-chain family member 1, promotes hepatic TG deposition and increased cholesterol production (12). However, the mechanisms of miRNAs associated with FLS in laying hens remain unclear. Hence, further explorations of these molecular mechanisms will be helpful for treatment of FLS and even provide important data for the future direction of treatments for patients with NAFLD, since chicken fatty liver is considered a good model of human NAFLD (13–15).

Therefore, the purpose of this study was to clarify the expression profiles of miRNAs associated with FLS in laying hens. The results showed that miR-216a/b and miR-217-5p, which belong to the miR-216/miR-217 cluster, were up-regulated in the sera and liver of a fatty liver chicken model. In addition, miR-216a/b and miR-217-5p, were found to inhibit expression of 3-hydroxyacyl-CoA dehydratase 2 (*HACD2*), F-box protein 8 (*FBXO8*), and transmembrane 9 superfamily member 3 (*TM9SF3*), respectively. Furthermore, *in vitro* studies demonstrated that overexpression of the miR-216/miR-217 cluster in LMH cells promoted hepatic steatosis *via* regulation of the PPAR/sterol regulatory-element binding protein (SREBP) pathway. These findings will help to clarify the roles of miRNAs in the pathogenesis of FLS in laying hens and NAFLD in humans.

## Materials and Methods

### Ethical Statement

The study protocol was approved by the Ethics and Animal Welfare Committee of the Shanghai Academy of Agricultural Sciences (Shanghai, China) and performed in accordance with the Guide for the Care and Use of Laboratory Animals as approved by the Ministry of Science and Technology of the People's Republic of China [Approval No. (2006) 398].

### Construction of a Fatty Liver Chicken Model

Hy-line Brown laying hens were raised under standard commercial conditions with *ad libitum* access to water as described in our previous study (16), and fed a corn-soy diet containing 16.0% crude protein and 2,700 kcal/kg of metabolizable energy. To identify the miRNAs differentially expressed between normal and fatty livers, 15 laying hens were killed at the ages of 25 and 52 weeks, respectively, and liver samples were harvested to assess lipid accumulation and RNA expression levels of selected biomarkers. At the age of 25 weeks, livers that were dark red with no hemorrhaging were considered normal.

### Histological Analysis

Livers were embedded in paraffin, cut into sections, and stained with hematoxylin and eosin to assess the extent of lipid accumulation. ImageJ software (version 1.80, National Institutes of Health, Bethesda, MD, USA) was used to quantify lipid droplets.

### FA Composition

Lipids were extracted for FA analysis with a gas chromatograph (model no. 6890; Agilent Technologies, Inc., Santa Clara, CA, USA) coupled to a mass selective detector (model no. 5973; Agilent Technologies, Inc.). Subsequently, the target compounds (fatty acid methyl esters, FAMEs) were transesterified with HCl in methanol. FAs were identified based on retention times with reference FA standards (Supelco 37-Component FAME Mix; Supelco Inc. Bellefonte, PA, USA). Individual FAs were calculated from the peak areas relative to the total area (total FAs were set at 100%). Three to five individual livers were pooled for four biological replicates.

### Small RNA Sequencing Analysis

Chicken liver RNA was purified using TRIzol^®^ Reagent. Five individual livers were pooled for three biological replicates. Small RNA sequencing was performed as described previously (17). Briefly, total RNA was extracted from livers and qualified on an Agilent 2100 Bioanalyzer System (Agilent Technologies, Inc.). Small RNA libraries were constructed and sequenced using a Hiseq 4000 Sequencing System (Illumina, Inc.). After sequencing, the raw date were aligned and mapped to the *Gallus* reference genome (https://ftp.ensembl.org/pub/release-81/fasta/gallus_gallus/dna/) using Langmead and Salzberg (18) and compared to miRBase (Release 21; https://www.mirbase.org/) to identify mature miRNAs. Then, novel miRNAs were predicted by miRDeep2 (19) and RNAfold (20). Differentially expressed miRNAs were obtained using the R DESeq package (https://bioconductor.org/packages/release/bioc/html/DESeq2.html). The raw sequencing data were deposited to the Sequence Read Archive of the National Center for Biotechnology Information (Accession no. PRJNA776040).

### Target Gene Prediction

Target genes of the miRNAs were predicted with miRnada (http://www.microrna.org) and TargetScan (https://www.targetscan.org/vert_80/) software as previously described (17). The 3′ untranslated regions (UTRs) of all known *Gallus gallus* genes were download from http://asia.ensembl.org/Gallus_gallus/Info/Index. Kyoto encyclopedia of genes and genomes (KEGG) pathway enrichment analysis was performed with reference to the DAVID 6.8 bioinformatic database (https://david.ncifcrf.gov/).

### Cell Culture and Cell Transfection

Chicken hepatocellular carcinoma (HCC) LMH cells (ATCC, Manassas, VA, USA) or HEK 293T cells (ATCC, Manassas, VA, USA) were cultivated in DMEM/F12 medium (GIBCO - Life Technologies, Carlsbad, CA, USA) or DMEM (high glucose) medium (GIBCO - Life Technologies) both containing 10% FBS (GIBCO - Life Technologies), 1% penicillin and streptomycin at 37 °C.

LMH cells (1.5 × 10^5^/mL) were seeded in the wells of 6-well-plates. LMH cells were transfected with mimics, inhibitors, scrambled oligonucleotides, siRNA-HACD2, siRNA-FBOX8, siRNA-TM9SF3, and control siRNA (40 nM, 10 pmol/mL) using Lipofectamine® 2000 Transfection Reagent (Invitrogen, Carlsbad, CA, USA) in Opti-MEM medium. The oligonucleotides used in this study were synthesized by Sangon Biotech Co., Ltd. (Shanghai, China) and are listed in Supplementary Tables 1, 2. At 24 h post-transfection, the cells were collected for quantitative real-time polymerase chain reaction (RT-qPCR) or protein analysis. The primers for RT-qPCR analysis are listed in Supplementary Table 3. To establish an *in vitro* fatty liver cell model, LMH cells were cultured in the presence of 1 mM free fatty acids (FFAs), containing oleic acid and palmitic acid at a 2:1 volume ratio, for 24 h prior to use for the indicated assays.

### Oil-Red O Staining

Liver samples were frozen on dry ice, and cut into 8-μm-thick sections, which were stained with an Oil Red O Stain Kit (Beijing Solarbio Science and Technology Co., Ltd., Beijing, China) in accordance with the manufacturer's protocol. LMH cells were transfected with mimics of miR-216a/b and miR-217-5p or scrambled oligonucleotides (40 nmol/L), as described above. At 24 h post-transfection, the cells were collected, fixed with 4% paraformaldehyde solution for 30 min, stained with oil red O stain, as described above, and imaged under electron microscope (Nikon Corporation, Tokyo, Japan). Meanwhile, LMH cells cultured with 1 mM FFAs for 24 h were used as a positive control.

### TG Contents Assay

The TG contents of culture media of LMH cells transfected with either an miRNA mimic or control for 24 h and liver tissues isolated from FLS chickens were measured using a commercial TG assay kit (Nanjing Jiancheng Bioengineering Institute, Nanjing, China) according to the manufacturer's instructions and normalized to the total protein concentration. The TG contents of the culture media and liver tissues are expressed as nmol/mL and mmol/μg protein, respectively. The culture medium of FFA-treated LMH cells was used as a positive control.

### Dual Luciferase Reporter Assay

Wild-type and mutated sequences of the 3′ UTR of the target miRNA (miR-216a/b and miR-217-5p) binding sites were synthesized and cloned into the plasmid psiCHECK-2 (Promega Corporation, Madison, WI, USA). Recombinant plasmids were co-transfected with miRNA mimic or scrambled control miRNA into HEK293T cells as described above. At 30 h post-transfection, luciferase activity was detected using the Dual-Glo^®^ Luciferase Assay System (Promega Corporation) in accordance with the manufacturer's instructions.

### RT-qPCR Analysis

Chicken liver, serum, and cell RNA was purified using TRIzol^®^ Reagent (Invitrogen). Amplification of mRNA for expression analysis was performed with SYBR Premix Ex Taq polymerase (Takara Bio, Inc., Shiga, Japan) using an ABI Q5 Real-time PCR System (Applied Biosystems, Foster City, CA, USA) with glyceraldehyde-3-phosphate dehydrogenase as internal references. The miRNA was reverse-transcribed into cDNA using the miScript II RT Kit (QIAGEN GmbH, Hilden, Germany) and amplified by RT-qPCR using a miScript SYBR Green PCR Kit (QIAGEN GmbH) with an ABI Q5 Real-time PCR System (Applied Biosystems). The miScript primers for selected miRNAs are the property of Qiagen. U6 was used as the internal control.

### Western Blot Analysis

Total protein was homogenized with radioimmunoprecipitation assay buffer (Roche Diagnostics GmbH, Mannheim, Germany), separated by electrophoresis using 8–10% sodium dodecyl sulfate gels, and transferred to polyvinylidene fluoride membranes (EMD Millipore Corporation, Billerica, MA, USA), which were blocked with 10% skim milk for 2 h and then probed with antibodies against HACD2 (bs-4429R; Bioss, Inc., Woburn, MA, USA), FBXO8 (bs-16053R; Bioss, Inc.), TM9SF3 (bs-19944R; Bioss, Inc.), and β-actin (8227; Abcam, Cambridge, MA, USA) and visualized with an enhanced chemiluminescence kit (WBKLS0050; EMD Millipore Corporation). The bands were imaged using a chemiluminescence imaging system (Syngene, Frederic, MD, USA).

### Statistical Analysis

The data were analyzed with a dependent sample t-test when the data of two groups conformed to a normal distribution, otherwise the nonparametric Mann-Whitney test was used. The data of three or more groups were analyzed by one-way analysis of variance and Tukey's HSD comparisons using SPSS 16.0. Results were presented as the mean ± SEM. A *P* < 0.05 was considered statistically significant.

## Results

### Hepatic Lipid Accumulation and FA Composition

**Figure 1:**
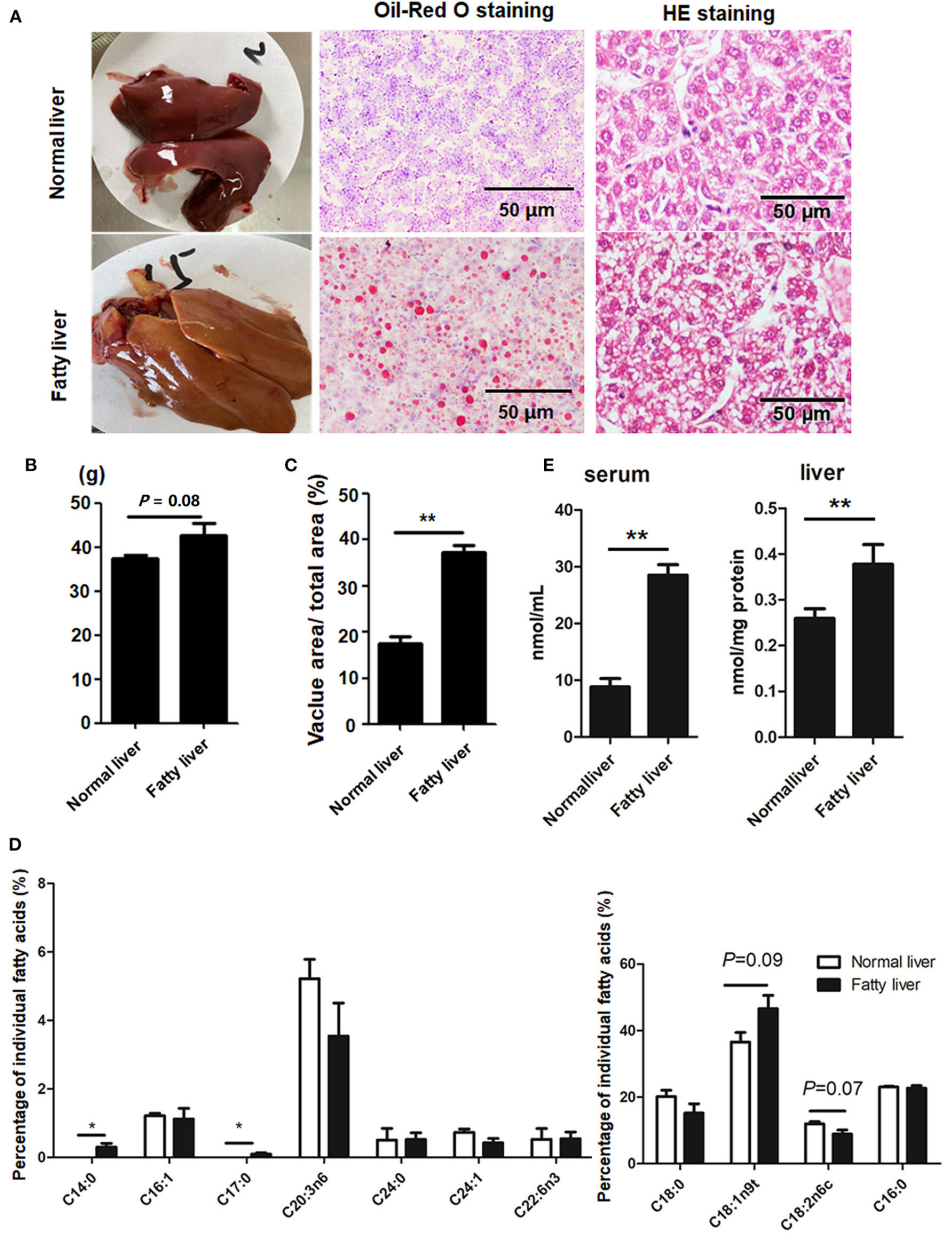
Hepatic lipid accumulation in chicken fatty liver **(A)** Representative images of H&E staining and Oil-Red O staining (200× magnification). **(B)** Weight of normal and fatty livers (*n* = 15). **(C)** Quantification results of fat vacuoles within the section. **(D)** FA composition between normal and fatty livers. Three to five individual livers were pooled for four biological replicates. The percentage of individual FAs was calculated according to the peak areas relative to the total area (total FAs were set at 100%). **(E)** Serum and Liver TG concentrations. *P < 0.05 and **P < 0.01.

Representative images of the pathological changes to the livers of chickens in the experimental and control groups at the ages of 25 and 52 weeks are presented in Figure 1A. At the age of 25 weeks, normal livers were dark red with no hemorrhaging. The livers of laying hens at 52 weeks of age were fragile and yellow in color with some hemorrhagic spots due to high lipid accumulation (Figure 1A) and, thus, were considered as fatty livers. The average weight of the fatty livers was relatively bigger than that of the normal livers (*P* = 0.08), while the vacuolar area was greater in the fatty livers as compared to the normal livers (*P* < 0.01) (Figures 1B,C). The amount of saturated FAs (C14:0 and C17:0) was significantly greater in the fatty livers as compared to the normal livers, while the amount of unsaturated FAs (C18:2n6c), especially omega-3 FAs, was relatively decreased in the fatty livers, but this difference was not statistically significant (*P* = 0.07, Figure 1D). Serum and liver TG contents were markedly increased in the fatty livers (Figure 1E, *P* < 0.05).

### miRNA Profiles of Normal and Fatty Livers

**Figure 2:**
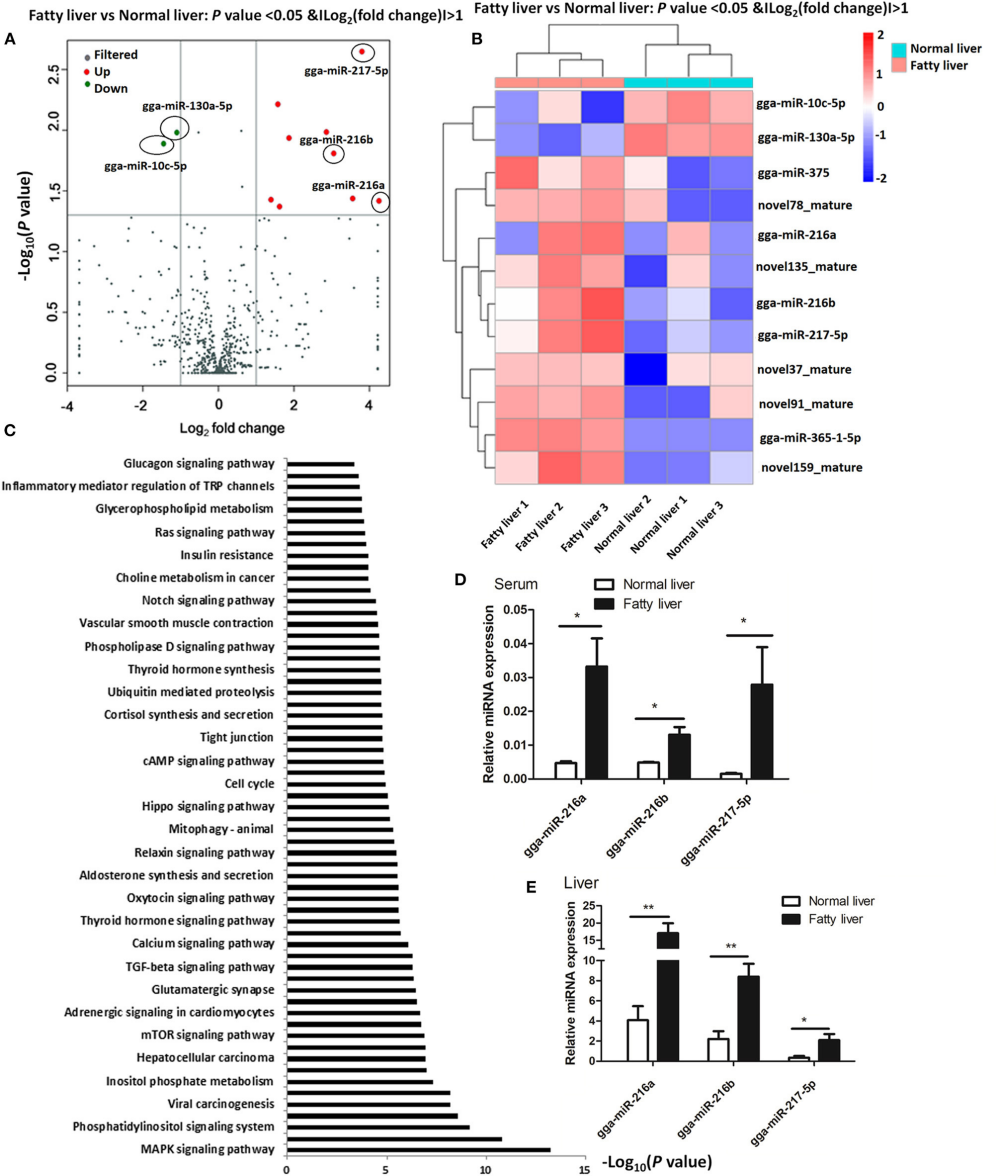
Identification of dysregulated miRNAs in chicken fatty liver **(A)** Volcano plot of miRNAs in normal and fatty livers. Significantly up-regulated miRNAs are shown in red and significantly down-regulated miRNAs in green. **(B)** Heat map of miRNAs in normal and fatty livers. **(C)** Function enrichment analysis of biological pathways for target genes of the 12 modulated miRNAs (fold change > 2). **(D,E)** Expression levels of selected miRNAs in sera and livers. *P < 0.05 and **P < 0.01.

As shown in Figures 2A,B, there was a >2-fold difference in 12 miRNAs between the normal and fatty livers collected at 25 and 52 weeks of age (Supplementary Table 4). Of these, 10 miRNAs (miR-216a/b, miR-217-5p, miR-375, miR-365-1-5p, novel91_mature, novel78_mature, novel159_mature, novel37_mature, and novel135_mature) were up-regulated and two (miR-10c-5p and miR-130a-5p) were down-regulated. Notably, miR-216a/b and miR-217-5p belong to the miR-216/miR-217 cluster.

The potential targets of the dysregulated miRNAs were predicted using miRnada and TargetScan software. KEGG pathway analysis showed that 31 pathways associated with the predicted miRNA targets were significantly enriched (Figure 2C and Supplementary Figure 1). Specifically, miRNA targets associated with the differentially expressed miRNAs belonged to multiple pathways, including phosphatidylinositol signaling system, cGMP-PKG signaling pathway, inositol phosphate metabolism, and pathways regulating lipid metabolism, such as glycerophospholipid metabolism, thyroid hormone synthesis, and insulin resistance (Figure 2C). In addition, as shown in Table 1, miR-216a was found to target synaptojanin 1 (*SYNJ1*) and *HACD2*, while miR-216b was shown to target *FBXO8* and ethanolamine kinase 1 (*ETNK1*), and miR-217-5p was shown to target regulating synaptic membrane exocytosis 2 (*RIMS2*) and *TM9SF3*.

**Table 1:** Potential targets of the miR-216/217 cluster.

**miRNA**	**Target score**	**Gene**	**Function**
gga-miR-216a	94	*TOB1*	Negative regulation of cell proliferation; tumor suppressor
	93	*SYNJ1*	Phosphatidylinositol phosphate metabolism
	93	*HACD2/(PTPLB)*	Sphingolipid biosynthetic process; very long-chain FA biosynthetic process
	91	*NAA15*	N-terminal protein amino acid acetylation; angiogenesis;cell differentiation
	91	*NADKD1*	NADP biosynthetic process; phosphorylation
	89	*LNPEP*	Insulin-mediated glucose transport
gga-miR-216b	97	*FBXO8*	Correlate with poor survival in hepatocellular carcinoma
	92	*ETNK1*	Plasmalogen biosynthesis
	86	*SERBP1*	Apoptosis-related network due to altered Notch3 in ovarian cancer
	84	*ZNF423*	TGF-β receptor signaling
gga-miR-217-5p	100	*RIMS2*	Cell differentiation; insulin secretion; Rab GTPase binding
	100	*MIER3*	Suppress cancer progression; tumor suppressor
	100	*YAF2*	Negative regulation of G0 to G1 transition; cell cycle
	99	*FBN2*	Sequestering of TGF-β in extracellular matrix
	99	*TM9SF3*	Participate in tumor invasion
	98	*PPM1D*	G2/M transition of mitotic cell cycle

Based on fold change and expression abundance, miR-216a/b and miR-217-5p were selected for further RT-qPCR analysis of the expression levels in sera samples and normal and fatty liver tissues specimens. The three miRNAs produced acceptable and consistent signals in the liver and sera samples of obese and normal control chickens. As shown in Figures 2D,E, the expression levels of miR-216a/b and miR-217-5p were relatively increased in obese chickens versus healthy controls with significant differences in miR-216a and miR-217-5p (>4-fold) (Figure 2).

### The miR-216/miR-217 Cluster Was Up-Regulated in FFA-Induced Fatty Liver Specimens

**Figure 3:**
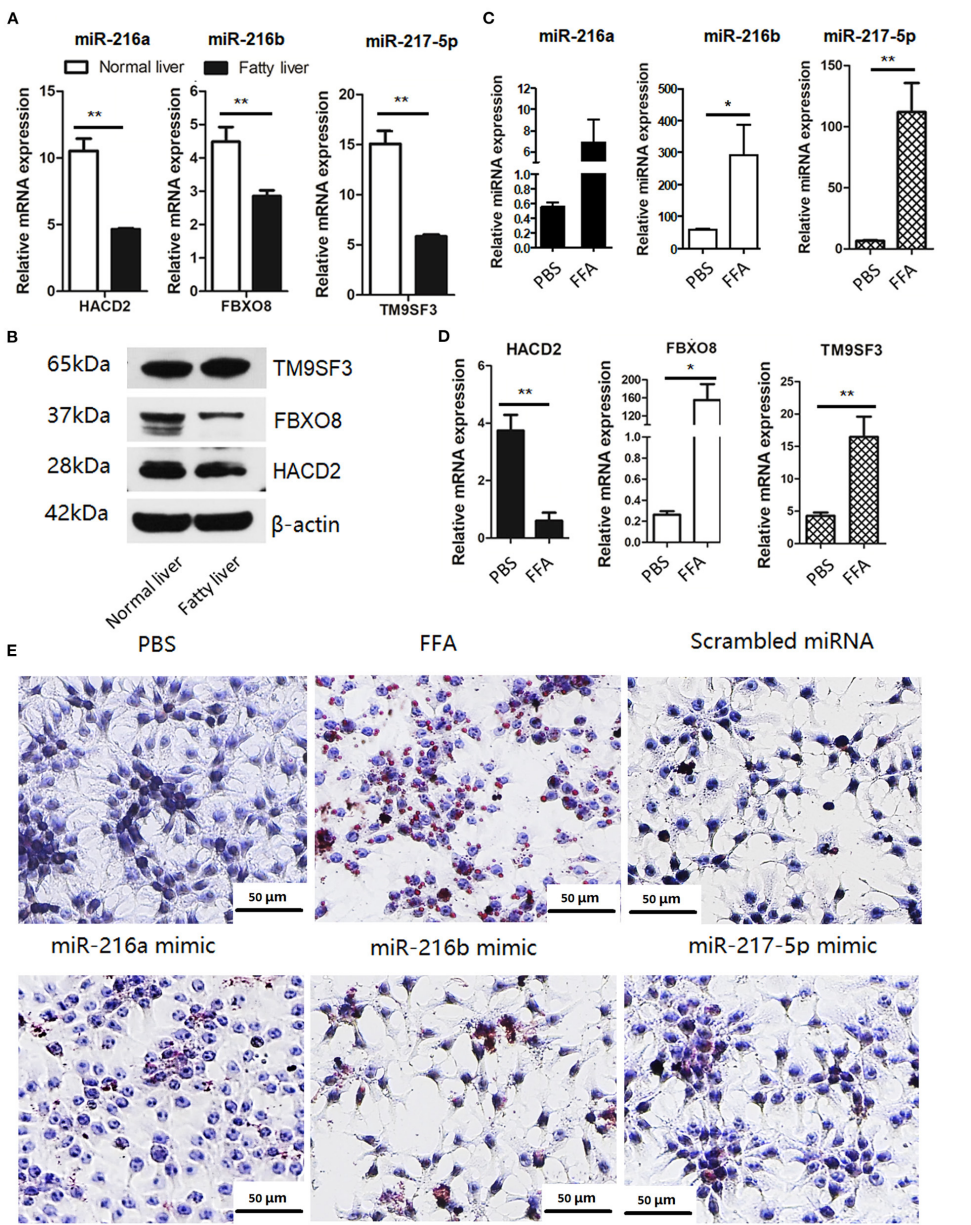
The miR-216/miR-217 cluster was up-regulated in FFA-induced f fatty liver model **(A)** mRNA levels of potential targets of the *in vivo* fatty liver model. **(B)** Protein levels of potential targets of the *in vivo* fatty liver model. **(C)** miRNA levels of the miR-216/217 cluster in FFA-induced fatty liver model. **(D)** mRNA levels of potential targets in FFA-induced fatty liver model. **(E)** Oil-red O staining (200× magnification). *P < 0.05 and **P < 0.01.

Based on the target gene predictions by miRnada and TargetScan software, the mRNA and protein expression levels of potential targets of miR-216a/b and miR-217-5p in the normal and fatty livers at 25 and 52 weeks of age, respectively, were analyzed by RT-qPCR. As shown in Figure 3A, the mRNA and protein expression levels of *HACD2* (potential target of miR-216a) and *FBXO8* (potential target of miR-216b) were comparatively down-regulated in the fatty livers, while mRNA expression of *TM9SF3* (potential target of miR-217-5p) was down-regulated in the fatty livers with no obvious difference in protein levels as compared to the normal liver (Figure 3B).

Furthermore, the miRNA levels of miR-216/miR-217 cluster and mRNA levels of the potential targets in FFA-induced fatty livers were analyzed. As shown in Figure 3, FFAs induced up-regulation of miR-216a/b and miR-217-5p in chicken LMH hepatocytes (Figure 3C) and decreased mRNA expression of *HACD2*, but not *FBXO8* and *TM9SF3* (Figure 3D). In addition, up-regulation of miR-216a/b or miR-217-5p induced lipid accumulation in LMH cells (Figure 3E), although there was no significant increase in TG content in the culture media of cells treated with the miRNA mimics, PBS, and control miRNAs (Supplementary Figure 2). These findings suggest that miR-216a/b and miR-217-5p may be involved in lipid metabolism.

### The miR-216/miR-217 Cluster Directly Targets HACD2, FBXO8, and TM9SF3

**Figure 4:**
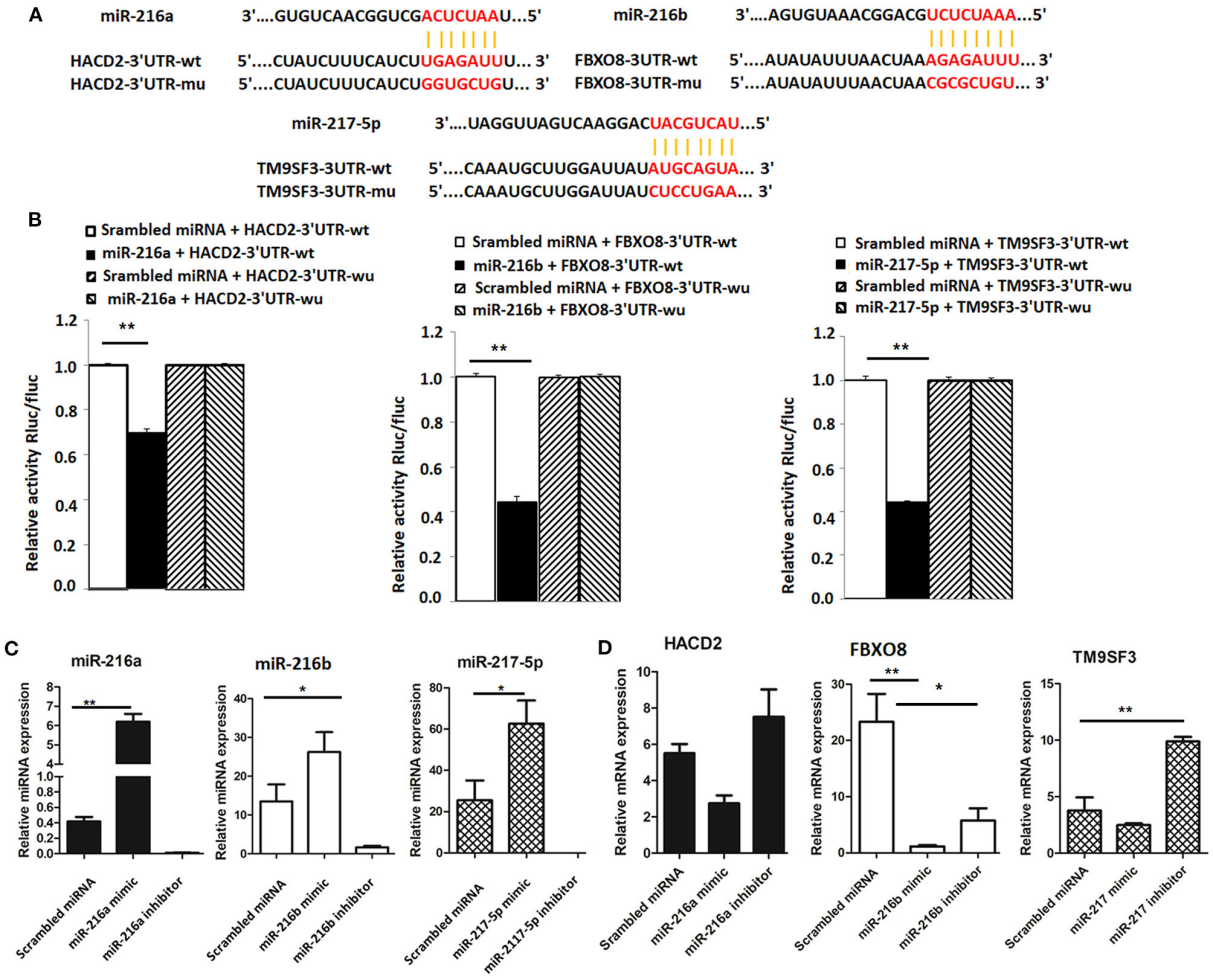
Validation of targets of the miR-216/miR-217 cluster **(A)** Wild-type and mutant sequences of *HACD2, FBXO8*, and *TM9SF3* **(B)** results of the dual luciferase reporter gene assay. **(C)** Expression of miR-216/miR-217 cluster members in LMH cells after up-regulation or down-regulation of the miR-216/miR-217 cluster. **(D)** mRNA levels of targets after up-regulation or down-regulation of the miR-216/miR-217 cluster. *P < 0.05 and **P < 0.01.

To determine whether *HACD2, FBXO8*, and *TM9SF3* are directly regulated by the miR-216/217 cluster, the capability of the miRNA mimics to inhibit luciferase activity was investigated in mammalian cells. The binding sites for *HACD2, FBXO8*, and *TM9SF3* mRNAs of the miR-216/miR-217 cluster were cloned into the 3′UTR of a luciferase reporter vector (Figure 4A). As shown in Figure 4B, transfection with the miR-216a/b and miR-217-5p miRNA mimics resulted in reduced luciferase activity as compared to transfection with the scrambled miRNA mimics. In contrast, the luciferase activity of the mutant-type 3′UTR was similar between the miRNA mimics and control mimics (Figure 4B).

Furthermore, to determine whether the miR-216/miR-217 cluster could inhibit mRNA expression of *HACD2, FBXO8*, and *TM9SF3 in vitro* in chicken liver cells, cultured LMH cells were transfected with mimics of miR-216a/b and miR-217-5p using Lipofectamine^®^ 2000 Transfection Reagent and changes to the mRNA expression levels of *HACD2, FBXO8*, and *TM9SF3* were assessed using RT-qPCR. As shown in Figure 4C, *in vivo* transfection with the miRNA mimics resulted in increased expression of miR-216a/b and miR-217-5p, while transfection with the miRNA inhibitors significantly inhibited expression of these miRNAs. As compared to the control group, the mRNA levels of *HACD2, FBXO8*, and *TM9SF3* were increased in the inhibitor groups and decreased in the mimic groups (Figure 4D). These results suggest that *HACD2* is directly targeted by miR-216a, *FBXO8* is a specific downstream target of miR-216b, and *TM9SF3* expression is directly regulated by miR-217-5p.

### Overexpression of the miR-216/miR-217 Cluster Regulates the PPAR/SREBP Signaling Pathway

**Figure 5:**
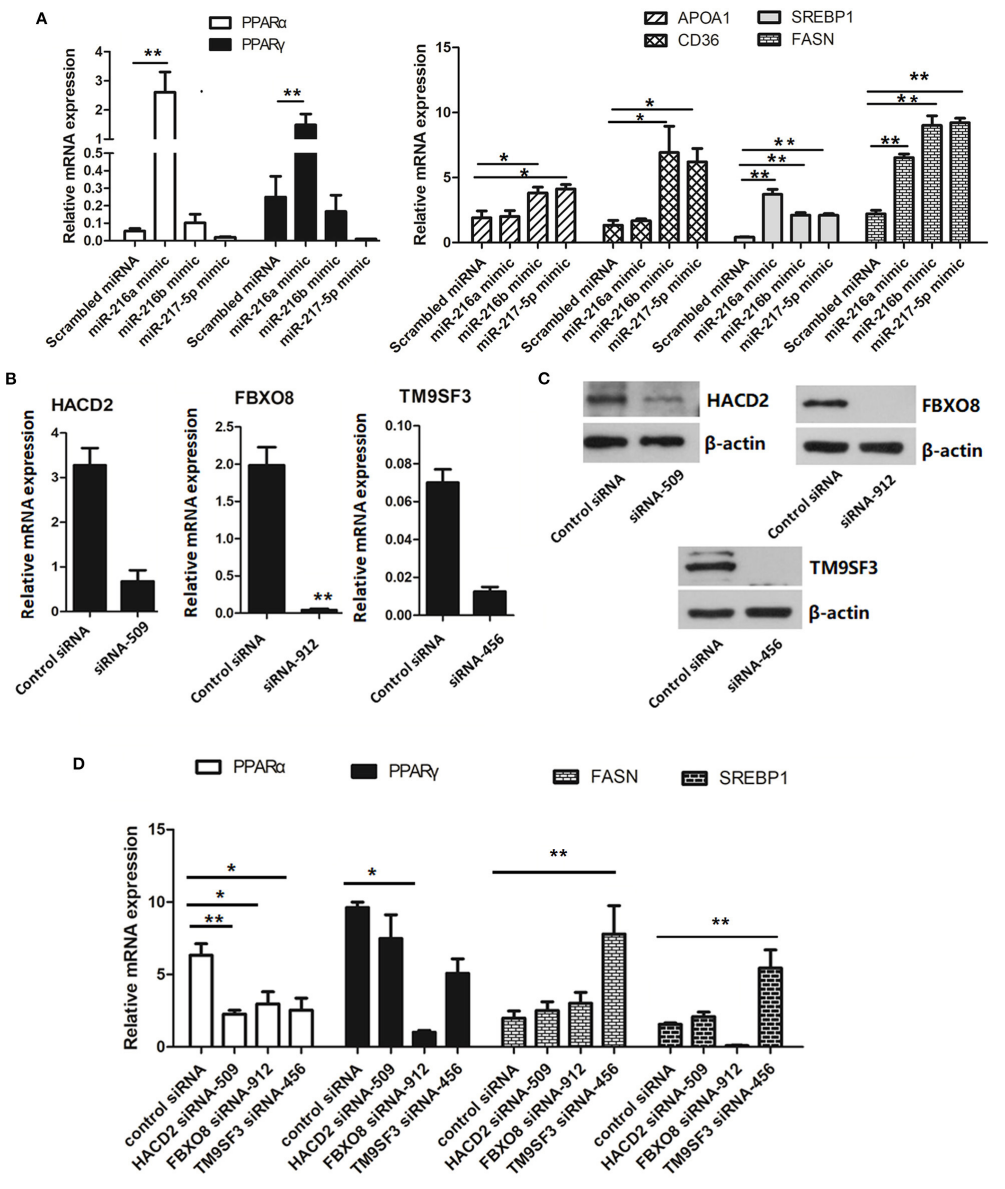
Relative mRNA levels of genes associated with lipid metabolism **(A)** relative mRNA levels of genes involved in the PPAR/SREBP signaling pathway in LMH cells transfected with miRNA mimics. **(B)** Validation of the siRNAs for silencing of *HACD2, FBOX8*, and *TM9SF3* by RT-qPCR analysis. **(C)** Validation of the siRNAs for silencing of *HACD2, FBOX8*, and *TM9SF3* by immunoblotting analysis. **(D)** RT-qPCR analysis of genes associated with the PPAR/SREBP signaling pathway in target-inhibited LMH cells. The results are presented as the mean and standard error of at least triplicate experiments. *P < 0.05 and **P < 0.01.

To further determine the regulatory role of the miR-216/miR-217 cluster in lipid metabolism, the mRNA expression levels of genes associated with the PPAR/SREBP signaling pathway were investigated. As shown in Figure 5A, overexpression of miR-216a significantly increased the mRNA levels of *PPAR*α and *PPAR*γ in cells transfected with the miR-217-5p mimic. Overexpression of miR-216b or miR-217-5p markedly enhanced the mRNA levels of hepatic *CD36* and apolipoprotein AI (*APOA1*) in LMH cells. The mRNA levels of the fat synthesis-related genes *SREBP1* and fatty acid synthase (*FASN*) were up-regulated in cells transfected with the miR-216a/b or miR-217-5p mimics.

To further confirm down-regulation of the PPAR/SREBP signaling pathway, siRNAs were designed and optimized to effectively silence *HACD2* (siRNA-509), *FBXO8* (siRNA-912), and *TM9SF3* (siRNA-456) (Figures 5B,C and Supplementary Figure 3). Treatment of LMH cells with the *TM9SF3* siRNA led to up-regulation of genes involved in the PPAR/SREBP signaling pathway, including *SREBP1* and *FASN*, but suppressed expression of *PPAR*α and *PPAR*γ (Figure 5D), which was consistent with miR-217-5p overexpression in LMH cells. Moreover, silencing of *HACD2* decreased mRNA expression of *PPAR*α. However, silencing of *HACD2* and *FBXO8* had no effect on *FASN* expression (Figure 5D). Overall, these results indicate that miR-217-5p induced downregulation of *TM9SF3* influenced the expression of genes involved in the PPAR/SREBP signaling pathway.

## Discussion

Although miRNAs have crucial regulatory effects on the pathogenesis of fatty liver disease (4–7), little is known about the specific molecular mechanisms of miRNAs in the regulation of FLS of laying hens. In this study, miRNA profile analysis identified 12 miRNAs that were significantly dysregulated in chicken fatty livers. Of the identified miRNAs, this study focused on miR-216a/b and miR-217-5p because the expression levels of both were consistently up-regulated in the sera and liver tissues of chickens with fatty livers and all three belong to the miR-216/217 cluster.

As reported in previous studies, miR-216a/b and miR-217-5p functions in obesity-related diseases, such as diabetes and NAFLD. miR-216a expression was increased during diabetes progression and essential for the proliferation of beta cells (21). The obesity-related gene phosphatase and tensin homolog was a direct target of miR-216a, which regulates expression of adiponectin receptor 1, caveolin-1, caveolin-2, and PPARγ (22, 23). Inhibition of miR-216b profoundly decreased the proliferation of HCC SMMC-7721 cells by regulating insulin-like growth factor 2 mRNA-binding protein 2, while overexpression of miR-216b inhibited replication of hepatitis B virus and proliferation of human hepatoblastoma HepG2.215 cells (24). Enhanced miR-216b-5p inhibited protein expression of uridine diphosphate-glucuronyltransferase 2B, which is an important enzyme in the detoxification of a variety of endogenous and exogenous compounds in both human hepatoma HuH-7 cells and human liver cancer Hep3B cells (25). Up-regulation of miR-217 in alpha mouse liver 12 cells promoted ethanol-mediated impairment of SIRT1 expression and FA oxidation enzymes (26). Here, high levels of miR-216a/b and miR-217-5p were observed in chicken fatty livers and FFA-induced fatty liver cells, suggesting that miR-21a/b and miR-217-5p may be involved in lipid metabolism.

FAs are the main components of lipids. HACD2, also called as protein tyrosine phosphatase-like member B (PTPLB), catalyzes the third step (dehydration) in the conversion of long-chain FAs to very long-chain FAs (27). Disruption of HACD2 significantly reduced elongation of both saturated and unsaturated FAs in the haploid human cell line HAP1 (27). In the present study, HACD2 was down-regulated in chicken fatty livers and the FFA-induced NAFLD model. In addition, the ratio of saturated FAs (C14:0 and C17:0) was dramatically increased, while that of unsaturated FAs (C18:2n6c) was relatively decreased in chicken fatty liver, which might be associated with down-regulation of HACD2. Moreover, miR-216a was found to possess binding sites for HACD2. Transfection with the miR-216a mimic reduced *HACD2* expression, while transfection with the miR-216a inhibitor had the opposite effect, suggesting that the diversity of FAs between normal and fatty livers may be regulated by miR-216a via targeting of *HACD2*. Additionally, miR-216b was found to target *FBXO8* and miR-217-5p targeted *TM9SF3*. Previous studies demonstrated that FBXO8 is lost in several cancers and associated with invasiveness of cancer cells, and decreased expression of *FBXO8* was correlated with poor survival of HCC patients, suggesting that FBXO8 is a potential biomarker of HCC progression (28). TM9SF3 is a nine-transmembrane protein that participates in tumor invasion and serves as a prognostic factor (29). TM9SF3 was also associated with insulin secretory granules, which are critical for the storage and secretion of insulin, although the detailed regulatory mechanism remains unclear (30). The down-regulation of *HACD2, TM9SF3*, and *FBXO8* in chicken fatty livers and LMH cells transfected with the miR-216/miR-217 cluster mimics suggests potential roles in the progression of FLS.

Hepatic lipid deposition, which is tightly controlled by key enzymes, including PPARα, PPARγ, CD36, APOA1, SREBP1, and FASN, involves FA synthesis, uptake, oxidation, and secretion (31). In particular, SREBP1 and FASN regulate lipogenesis (31, 32), while CD36 facilitates cellular uptake and intracellular trafficking of FAs (33). Enhanced expression of CD36 was reported to promote hepatic FA uptake and lipid deposition both *in vivo* and *in vitro* (33). In addition, CD36 is a shared target of PPARγ (34). PPARα and PPARγ are members of the nuclear receptor superfamily involved in hepatic β-oxidation, lipid storage, and glucose homeostasis (35). Here, transfection of LMH cells with miR-216/miR-217 cluster mimics regulated the expression of genes associated with the PPAR/SREBP signaling pathway. Notably, overexpression of miR-217-5p or inhibition of *TM9SF3* down-regulated expression of the FA oxidation-related genes *PPAR*α and *PPAR*γ, but up-regulate the lipogenesis-related genes *FASN* and *SREBP1*. Taken together, these results suggest that the miR-216/miR-217 cluster can regulate lipid metabolism.

Notably, previous studies showed that miR-216a/b and miR-217-5p were expressed in different disease models. For example, miR-216a/b and miR-217-5p were decreased in HCC (24, 36, 37), while Greco et al. (38) showed that up-regulation of miR-216a was linked to diabetic heart failure. Up-regulation of the miR-216a/217 cluster was observed in HCC tissue samples and cell lines, which were also found to be responsible for early tumor recurrence (39). Higher insulin production was observed in an animal model of type 1 diabetes treated with a nanodrug carrying the miR-216a mimic, as compared to untreated controls (21). Moreover, miR-217 was upregulated in sorafenib-resistant HCC cells and hepatitis B virus-associated HCC (40, 41). Here, the miR-216/miR-217 cluster was up-regulated in chicken fatty livers and FFA-induced LMH cells. Collectively, these results demonstrate that the function of the miR-216/miR-217 cluster varies in different disease models. Thus, further studies are warranted to discern the function of the miR-216/miR-217 cluster in specific diseases. Additionally, hepatic lipid metabolism in laying hens is a relatively complex process. The specific function of the miR-216/miR-217 cluster in different production stages of laying hens still needs further investigation, since the hepatic lipid metabolism is strongly activated in liver of hen with sex maturation, but dysregulated in FLS laying hens (42).

## Conclusion

In conclusion, we demonstrated that hepatic miR-216a/b and miR-217-5p levels increased in FLS and that the miR-216/miR-217 cluster is involved in lipid metabolism via targeting *HACD2, FBXO8*, and *TM9SF3*. Furthermore, overexpression of the miR-216/miR-217 cluster activated the PPAR/SREBP signaling pathway. These findings provide new insights into the roles of miRNAs in fatty liver diseases and may contribute to the development of novel strategies for the treatment of NAFLD and FLS.

## Data Availability Statement

The datasets presented in this study can be found in online repositories. The names of the repository/repositories and accession number(s) can be found in the article/Supplementary Material.

## Ethics Statement

The animal study was reviewed and approved by the Ethics and Animal Welfare Committee of the Shanghai Academy of Agricultural Sciences (Shanghai, China) and performed in accordance with the Guide for the Care and Use of Laboratory Animals as approved by the Ministry of Science and Technology of the People's Republic of China [Approval No. (2006) 398].

## Author Contributions

CY and HW supported the funding. RL performed the cell culture, cell transfection, and oil red O staining experiment. JH did the RT-qPCR experiment. HY, CX, and YY collected the samples. LZ designed the experiments, analyzed the data, performed the experiments, and wrote the manuscript. All authors read and approved the final manuscript.

## Funding

This work was supported by China Agriculture Research System under Grant (CARS-40-K03), SAAS Program for Excellent Research Team (Grant Number 2022-021), and Climbing Plan of Shanghai Academy of Agricultural Sciences (PG21171).

## Conflict of Interest

The authors declare that the research was conducted in the absence of any commercial or financial relationships that could be construed as a potential conflict of interest.

## Publisher's Note

All claims expressed in this article are solely those of the authors and do not necessarily represent those of their affiliated organizations, or those of the publisher, the editors and the reviewers. Any product that may be evaluated in this article, or claim that may be made by its manufacturer, is not guaranteed or endorsed by the publisher.
