# Supplementary material for: The miR-216/miR-217 Cluster Regulates Lipid Metabolism in Laying Hens With Fatty Liver Syndrome via PPAR/SREBP Signaling Pathway
Source: Front Vet Sci. 2022 May 31;9:913841. doi: 10.3389/fvets.2022.913841 (PMC9195098; doi:10.3389/fvets.2022.913841)
Supplement: Supplementary file 1 [file Table_1.DOCX]

**Supplementary Tables:**

**Supplementary Table S1** List of miRNA mimics/anti-sense miRNA used in the study.

| **Gene** | **Sence（5'-3'）** | **Antisence（5'-3'）** |
| --- | --- | --- |
| miR-216a mimic | UAAUCUCAGCUGGCAACUGUG | CAGUUGCCAGCUGAGAUUAUU |
| miR-217-5p mimic | UACUGCAUCAGGAACUGAUUGGAU | CCAAUCAGUUCCUGAUGCAGUAUU |
| miR-216b mimic | AAAUCUCUGCAGGCAAAUGUGA | ACAUUUGCCUGCAGAGAUUUU |
| Scrambled miRNA | UUGUACUACACAAAAGUACUG | GUACUUUUGUGUAGUACAAUU |
| miR-216a inhibitor | CACAGUUGCCAGCUGAGAUUA |  |
| miR-217-5p inhibitor | AUCCAAUCAGUUCCUGAUGCAGUA |  |
| miR-216b inhibitor | UCACAUUUGCCUGCAGAGAUUU |  |

**Supplementary Table S2.** List of siRNAs for miRNA target silencing in the study

| **Gene IDs** | **siRNA names** | **Sequence（5'-3'）** |
| --- | --- | --- |
| *HACD2* | HACD2-siRNA-431 | Sense GGACAAUCACAGAGAUAAUTT |
|  |  | Antisense AUUAUCUCUGUGAUUGUCCTT |
|  | HACD2-siRNA-509 | Sense CCAGGUACACUUUGUUUAUTT |
|  |  | Antisense AUAAACAAAGUGUACCUGGTT |
|  | HACD2-siRNA-743 | Sense CCCACACUGAAGAACACAATT |
|  |  | Antisense UUGUGUUCUUCAGUGUGGGTT |
| *FBXO8* | FBXO8-siRNA-230 | Sense CCAGCAACUGCAACACCAATT |
|  |  | Antisense UUGGUGUUGCAGUUGCUGGTT |
|  | FBXO8-siRNA-289 | Sense GGAGAAUAGCUGCUAACAATT |
|  |  | Antisense UUGUUAGCAGCUAUUCUCCTT |
|  | FBXO8-siRNA-912 | Sense UCUCACAGAUUCUGUGCUUTT |
|  |  | Antisense AAGCACAGAAUCUGUGAGATT |
| *TM9SF3* | TM9SF3-siRNA-456 | Sense GGACAAAGGAAGAAGAGAATT |
|  |  | Antisense UUCUCUUCUUCCUUUGUCCTT |
|  | TM9SF3-siRNA-1171 | Sense GGAGGAAGGCGGUGGAUAATT |
|  |  | Antisense GGAGGAAGGCGGUGGAUAATT |
|  | TM9SF3-siRNA-1839 | Sense GGGAACAAGUGCCUUUGUUTT |
|  |  | Antisense AACAAAGGCACUUGUUCCCTT |
| Control siRNA |  | Sense UUCUCCGAACGUGUCACGUTT |
|  |  | Antisense ACGUGACACGUUCGGAGAATT |

**Supplementary Table S3.** List of the primers used for RT-qPCR analysis in the study

| **Gene** | **Forward primer （5-3）** | **Reverse primer （5-3）** |
| --- | --- | --- |
| *GAPDH* | GATGGGTGTCAACCATGAGAAA | CAATGCCAAAGTTGTCATGGA |
| *PPARα* | GCTTGTGAAGGTTGTAAGGGTT | GACATTCCAACTGAAAGGCAC |
| *PPARγ* | GTCCTTCCCGCTGACCAAA | TCTCCTGCACTGCCTCCACA |
| *CD36* | GCTTTGCAGGAGCTAATGTCTAG | CTGGGAAGGTTACTGCGATTT |
| *APOA1* | GTGACCCTCGCTGTGCTCTT | CTCAGCGTGTCCAGGTTGT |
| *SREBP1* | CAGAGCTGATGGAGGGCAT | TCTTCGTGGACGGGGATT |
| *FASN* | CTTCAGAGCGACAATACCCA | AAGCAATTCGTCACGGACA |
| *HACD2* | CCAAGTGATGTCAAGGGTTTT | ACTGCCTGACGAAGGGTAAT |
| *FBXO8* | AAATCCACTTGGGGTCACTG | AAGGTCATCCAAAACATCTCG |
| *TM9SF3* | TTTGACAAGTACCTTGACCCAT | AAAATCTGACACCCAGAGCC |
| *U6* | GGAACGATACAGAGAAGATTAGC | TGGAACGCTTCACGAATTTGCG |

**Supplementary Table S4.** List of miRNAs identified differentially expressed in fatty liver of laying hens

| **miRNA_id** | **Fold-change (log2 Fatty liver/Normal liver)** | ***P* value** | **Sequence** | **Length** | **miRNA family** |
| --- | --- | --- | --- | --- | --- |
| gga-miR-216a | 18.62 | 0.04 | TAATCTCAGCTGGCAACTGTG | 21 | miR-216 |
| gga-miR-217-5p | 13.97 | 0.00 | TACTGCATCAGGAACTGATTGGAT | 24 | miR-217 |
| novel91_mature | 12.01 | 0.04 | CGGCGCGCGGCTCGGCGC | 18 |  |
| novel78_mature | 8.16 | 0.02 | TTGGTGTCACTGCAGAGC | 18 |  |
| gga-miR-216b | 7.27 | 0.01 | AAATCTCTGCAGGCAAATGTGA | 22 | miR-216 |
| novel159_mature | 3.70 | 0.01 | GGCGCCGCCGGGCCGGGT | 18 |  |
| novel37_mature | 3.13 | 0.04 | AGCTGTGAGGCTTCCTCC | 18 |  |
| gga-miR-375 | 2.97 | 0.01 | TTTGTTCGTTCGGCTCGCGTTA | 22 | miR-375 |
| novel135_mature | 2.58 | 0.04 | TCCGGGCACGGTGCACCT | 18 |  |
| gga-miR-365-1-5p | 2.43 | 0.04 | GAGGGACTTTTGGGGGCAGATGT | 23 | miR-365 |
| gga-miR-130a-5p | 0.48 | 0.01 | GCCCTTTTTCTGTTGTACTACT | 22 | miR-130 |
| gga-miR-10c-5p | 0.37 | 0.01 | TACCCTGTAGACTCGAATTTGT | 22 | miR-10 |
