# Supplementary material for: The miR-216/miR-217 Cluster Regulates Lipid Metabolism in Laying Hens With Fatty Liver Syndrome via PPAR/SREBP Signaling Pathway
Source: Front Vet Sci. 2022 May 31;9:913841. doi: 10.3389/fvets.2022.913841 (PMC9195098; doi:10.3389/fvets.2022.913841)
Supplement: Supplementary file 2 [file Table_2.DOCX]

**Supplementary Figures:**

**
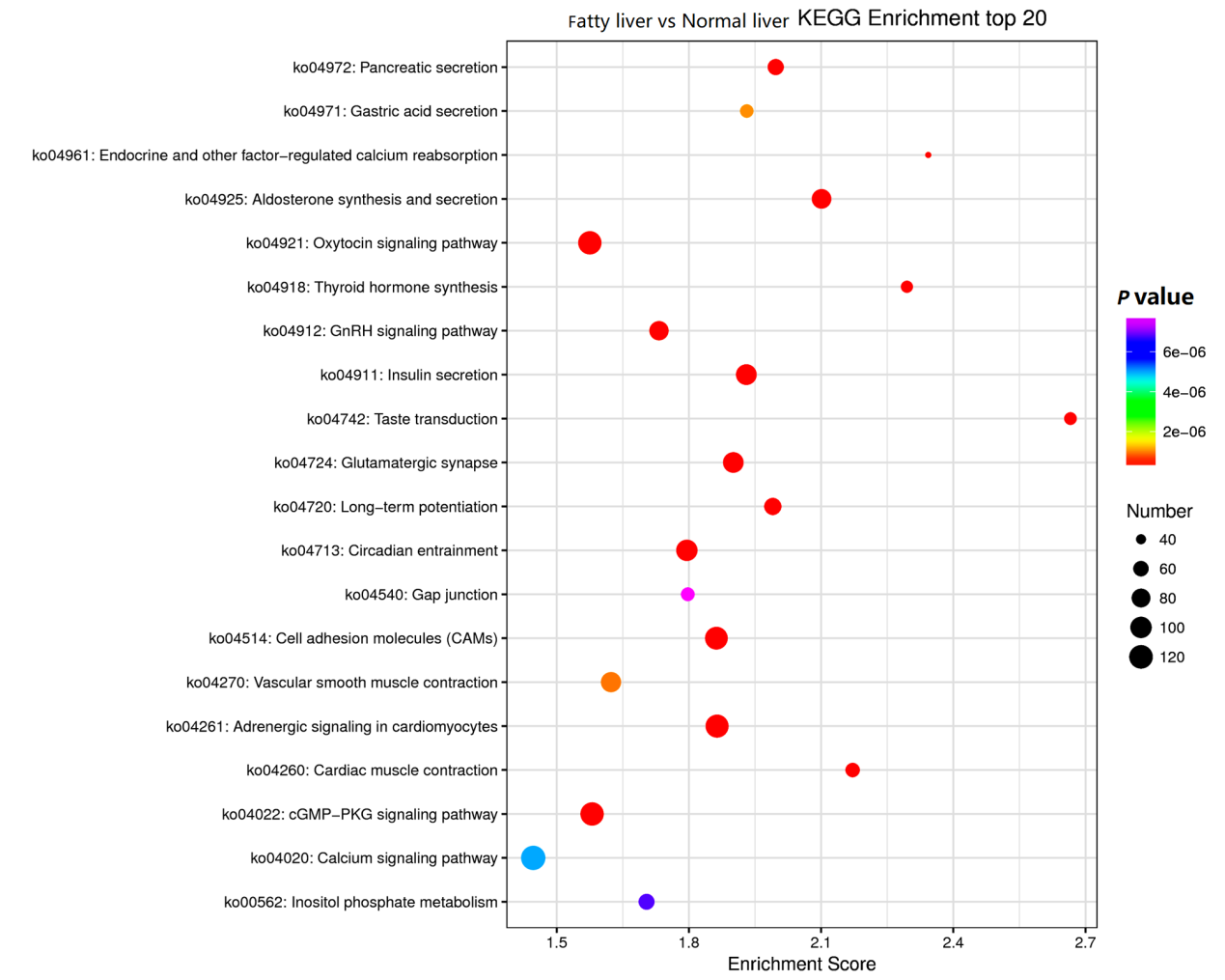
**

**FIGURE S1** KEGG pathways classification analysis for potential targets of differentially expressed miRNAs. **P <* 0.05 and ***P <* 0.01.


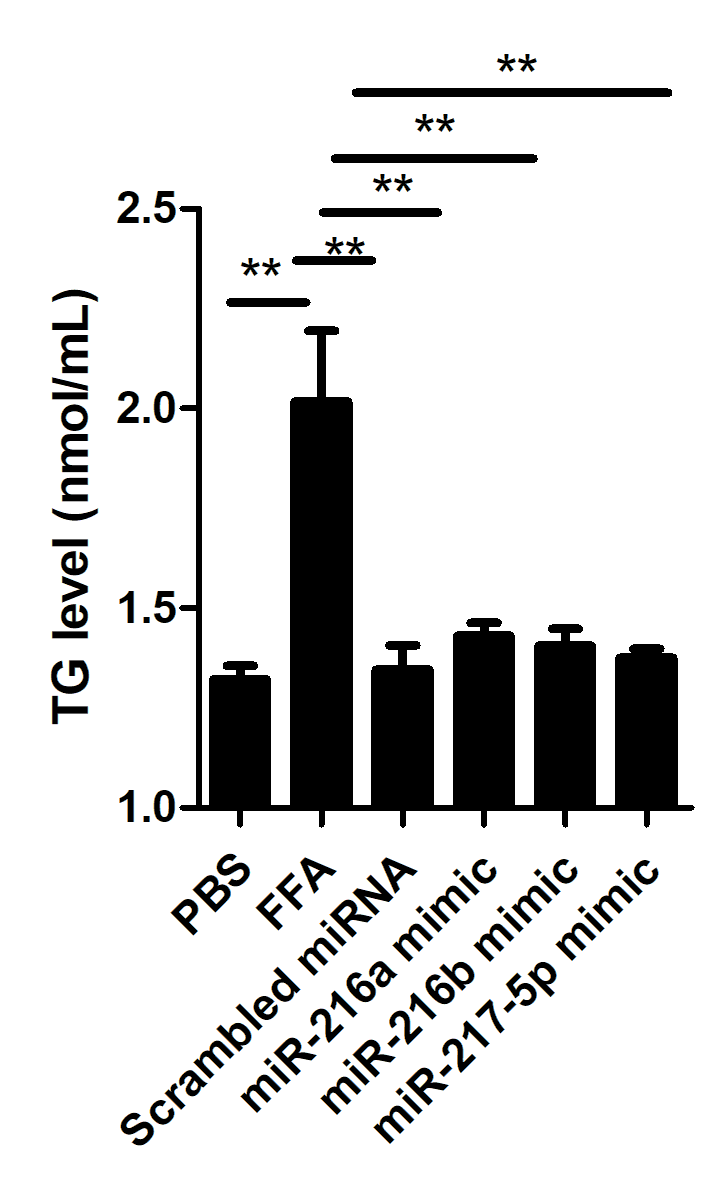


**FIGURE S2** Content of TG in the cell culture media of FFA-induced fatty liver and miRNA mimic transfected cells. **P <* 0.05 and ***P <* 0.01.


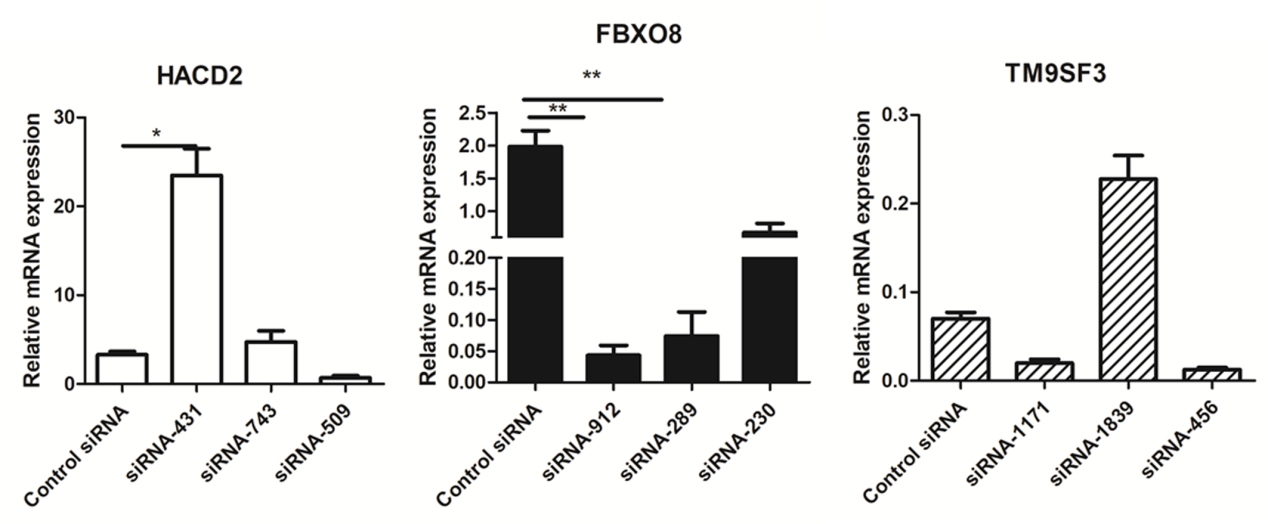


**FIGURE S3.** Selection of siRNA for inhibiting the expression of *HACD2*, *FBXO8*, and *TM9SF3*. **P <* 0.05 and ***P <* 0.01.
